# Would treatment decisions about secondary prevention of CVD based on estimated lifetime benefit rather than 10-year risk reduction be cost-effective?

**DOI:** 10.1186/s41512-020-00072-5

**Published:** 2020-04-16

**Authors:** Gijs F. N. Berkelmans, Jacoba P. Greving, Yolanda van der Graaf, Frank L. J. Visseren, Jannick A. N. Dorresteijn

**Affiliations:** 1 Department of Vascular Medicine, University Medical Center Utrecht, PO Box 85500, 3508 Utrecht, GA The Netherlands; 2 Julius Center, University Medical Center Utrecht, Utrecht, The Netherlands

**Keywords:** Lifetime benefit, Lifetime expectancy estimation, Cost-effectiveness, Prevention, PCSK9-mAb

## Abstract

**Objective:**

To test the hypothesis that treatment decisions (treatment with a PCSK9-mAb versus no treatment) are both more effective and more cost-effective when based on estimated lifetime benefit than when based on estimated risk reduction over 10 years.

**Methods:**

A microsimulation model was constructed for 10,000 patients with stable cardiovascular disease (CVD). Costs and quality-adjusted life years (QALYs) due to recurrent cardiovascular events and (non)vascular death were estimated for lifetime benefit-based compared to 10-year risk-based treatment, with PCSK9 inhibition as an illustration example. Lifetime benefit in months gained and 10-year absolute risk reduction were estimated using the SMART-REACH model, including an individualized treatment effect of PCSK9 inhibitors based on baseline low-density lipoprotein cholesterol. For the different numbers of patients treated (i.e. the 5%, 10%, and 20% of patients with the highest estimated benefit of both strategies), cost-effectiveness was assessed using the incremental cost-effectiveness ratio (ICER), indicating additional costs per QALY gain.

**Results:**

Lifetime benefit-based treatment of 5%, 10%, and 20% of patients with the highest estimated benefit resulted in an ICER of €36,440/QALY, €39,650/QALY, or €41,426/QALY. Ten-year risk-based treatment decisions of 5%, 10%, and 20% of patients with the highest estimated risk reduction resulted in an ICER of €48,187/QALY, €53,368/QALY, or €52,390/QALY.

**Conclusion:**

Treatment decisions (treatment with a PCSK9-mAb versus no treatment) are both more effective and more cost-effective when based on estimated lifetime benefit than when based on estimated risk reduction over 10 years

## Introduction

Recent guidelines for cardiovascular prevention all recommend estimating an individual patient’s risk (10-year risk of cardiovascular disease) for decision-making on whether or not to start preventive interventions [1–4]. The potential benefit of preventive treatment is often assessed with risk reduction over a fixed period. However, with chronically progressive diseases, the main aim of treatment is often to prolong the disease-free life expectancy [5]. The use of lifetime prediction models that adjust for competing risks provides a more intuitive approach which identifies younger patients who would benefit from treatment they would otherwise be denied and older patients who might not benefit from treatment they would otherwise be offered [6].

A recently developed prediction model for secondary prevention, the SMART-REACH model, is able to estimate individual benefit of medication for prevention of CVD in patients with a history of stable CVD as 10-year risk reduction or as months gained from a lifetime perspective, the lifetime benefit (supplemental methods) [7]. Estimation of treatment effects expressed by a lifetime benefit could overcome some disadvantages of the 10-year risk-based strategies. Younger patients with a low 10-year risk, but high risk factor levels, will have a high estimated lifetime benefit because lifetime prediction models take long-term exposure of risk factors and follow-up time into account [8]. On the other hand, in patients older than 70 years of age, the high estimated 10-year risk for fatal CVD may falsely suggest large estimated 10-year risk reduction of preventive treatment. As older patients are also at risk for non-CVD mortality, any reductions in CVD-mortality risk may be counterbalanced by a high risk for non-CVD mortality. This may result in 10-year risk estimations leading to an overestimation of the potential benefit of preventive treatment in older patients [9].

Although it is tempting to assume estimations from a lifetime perspective could be useful in the identification of patients that benefit most from preventive treatment and interventions, there is no evidence on the cost-effectiveness of lifetime benefit assessment for guiding pharmacological therapy decisions [1]. Also, starting preventive interventions at a younger age means longer treatment duration and therefore higher costs and more harm. Because modelling effectiveness of CVD prevention is complex, we opted to use a simplified model of secondary prevention of CVD with proprotein convertase subtilisin/kexin type 9 (PCSK9) inhibition treatment with monoclonal antibodies (mAbs) using a microsimulation model with a lifetime horizon [10]. PCSK9 inhibitors are a new class of drugs that effectively reduce low-density lipoprotein cholesterol (LDL-c) levels by 50–60% and reduce CVD [11–14].

We tested the hypothesis that treatment decisions (treatment with a PCSK9-mAb versus no treatment) are both more effective and more cost-effective when based on estimated lifetime benefit than when based on estimated risk reduction over 10 years.

## Methods

**Fig. 1:**
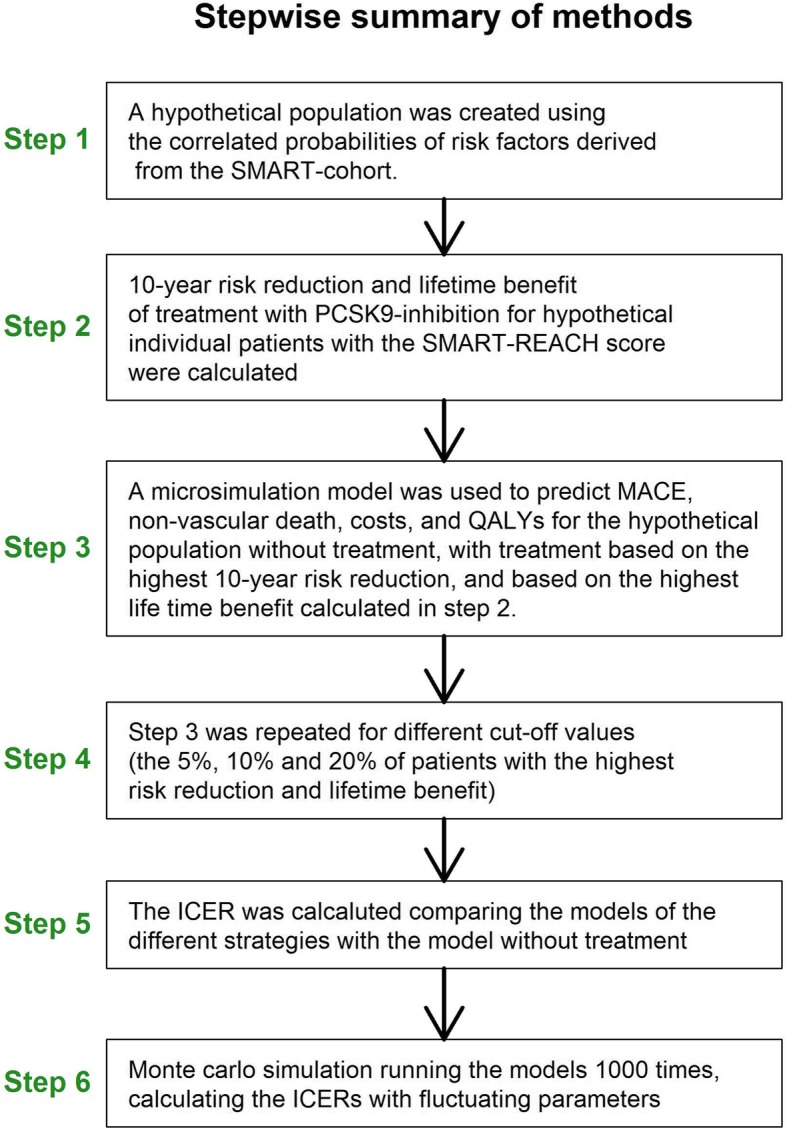
A brief explanation of the methods

A stepwise summary of the methods is shown in Fig. 1.

### Study population

In order to get a representation of a national population of patients with stable CVD, a hypothetical population was created by repeatedly sampling from correlated probability distributions of risk factors. A correlated probability distribution makes it possible to randomly sample a set of variables from a hypothetical population while preserving the correlations between the different variables. A simplified example: if the variable systolic blood pressure is randomly chosen to be 180 mmHg, the variable age will be sampled from a distribution with a higher mean—blood pressure and age are correlated, with higher blood pressures tending to be found with greater age. The correlated probability distribution used to create this hypothetical population was obtained from the Second Manifestation of ARTerial disease (SMART) cohort described elsewhere [15]. In brief, the SMART cohort consists of 7519 patients with clinical manifestations of vascular disease included between 1996 and 2015. Of these patients, baseline measurements of risk factors were performed using a standardized protocol. For our study, a population of 10,000 hypothetical patients was created by randomly sampling these baseline risk factor variables and the distribution among patients of age, sex, smoking, diabetes mellitus, systolic blood pressure, total cholesterol, LDL-c, creatinine, and number of locations with vascular disease. Baseline variables of atrial fibrillation and chronic heart failure were not available in the SMART cohort. Therefore, the sampling distributions of atrial fibrillation and chronic heart failure were established from literature and only correlated with age and sex [16, 17].

### Individual treatment effect estimations

Individualized 10-year risk reduction and lifetime benefit treatment effects of treatment with a PCSK9-mAb versus no treatment were estimated using the SMART-REACH model [7]. The SMART-REACH model is a model to estimate life expectancy free of a recurrent CVD in patients with a history of CVD. It is based on the competing risk model of Fine and Gray, and the age of patients is used as the time scale (left truncation) [18]. To estimate individual treatment effects of PCSK9-mAbs on recurrent CVD in this study, a coefficient based on the relative risk reduction reported by meta-analyses was added to the model.

Our assumption of the effect of PCSK9-mAbs was based on the expected LDL-c reduction, which is dependent on the baseline LDL-c level [13]. On average, PCSK9-mAbs have been shown to reduce LDL-c levels by 50–60% [14]. In the present study, a conservative estimate of treatment benefit of 50% LDL-c reduction was assumed. The results of the recent PSCK9 inhibitor outcome trial correspond with the more robust results from large meta-analyses showing a hazard ratio of 0.78 (95% CI 0.76–0.80) for major vascular events per 1 mmol/L LDL-c reduction [11, 12]. There was no indication of a decreasing effect size when LDL-c levels were reduced below 2 mmol/L [11]. Thus, for our study, the individualized relative treatment effect of PCSK9 inhibition on CVD based on expected LDL-c reduction was defined as 0.78^0.5*LDL-c^. Individualized hazard ratios (HRs) were calculated for each study participant. We assumed that LDL-c reduction has no effect on non-vascular mortality [11]. The lifetime benefit of treatment (number of CVD-free life-years gained by therapy) was calculated as the difference between the estimated life expectancy free of recurrent CVD with treatment and without treatment. The 10-year absolute risk reduction was calculated as difference between the expected 10-year risk with treatment and without treatment (supplemental methods).

### Microsimulation model design

**Fig. 2:**
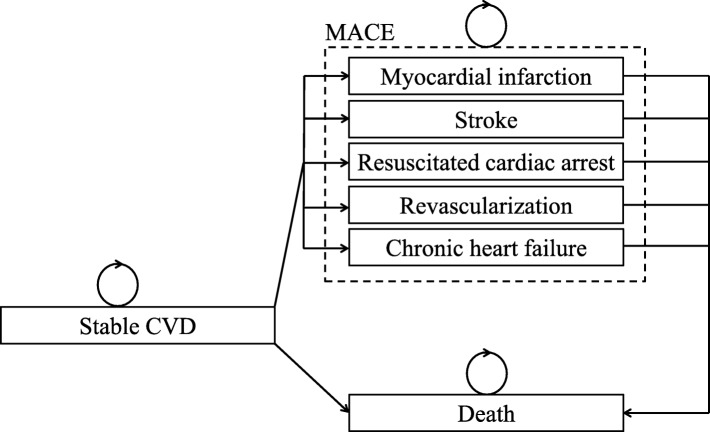
Diagrammatic representation of the micro-simulation model with health states (boxes) and possible transitions (arrows)

A microsimulation model was developed to predict major cardiovascular events (MACE), (non)vascular death, costs and quality-adjusted life years (QALYs) for risk-based treatment, and lifetime-benefit-based treatment, using treatment with PCSK9-mAbs as an example [10]. Expected outcome for patients within the study population with the highest predicted treatment effect based on 10-year absolute risk reduction and lifetime benefit were compared, using different cut-off values (i.e. the best 5%, 10%, and 20% of patients with the highest estimated benefit). The microsimulation model contained three health states: ‘stable cardiovascular disease’, ‘recurrent MACE’, and ‘death’. All hypothetical patients started in the ‘stable cardiovascular disease’ health state. Patients could stay in their health state or transit to another health state each year (Fig. 2). Patients transit to the recurrent MACE state if they experienced a MACE in the particular year, namely a myocardial infarction, ischemic stroke, or haemorrhagic stroke. Patients transit to the ‘death’ health state whenever they died of any cause and remained in that health state. The simulation ran until all hypothetical patients had died, i.e. for a lifetime horizon.

### Model variables

#### Transition risks

This economic evaluation was performed from a health care perspective, meaning that only medical and not societal costs and effects were evaluated. The probabilities of transition from the stable CVD health state to the MACE health state were based on mean annual cardiovascular event risks. Mean annual event risks for myocardial infarction, stroke, and revascularization without PCSK9 treatment were derived from the SMART cohort (supplemental table 1) [15]. Mean annual event risks for resuscitated cardiac arrest and heart failure without PCSK9 treatment were derived from the intensive treatment arm of the TNT trial (supplemental table 1) [19]. The individualized event risks changed with age according to an existing 10-year risk score for patients with CVD (supplemental figure 1), systolic blood pressure, current smoking, and diabetes mellitus [20]. The annual event rates were multiplied by the HR to obtain an individualized expected treatment effect when a patient was treated with PCSK9-mAbs. Case-fatality rates for myocardial infarction and stroke were age-dependent and obtained from the Dutch nationwide registries for in- and outside hospital deaths [21–23]. The probability of non-vascular death for patients with stable CVD or patients in the post-event health state was estimated by multiplying the age-adjusted probability of non-vascular death in the general population by a disease-specific mortality multiplier (supplemental table 1) [24–30].

#### Health outcomes

The number of life years and QALYs for each patient was estimated for the different treatment strategies (treatment of patients within the study population with the highest predicted treatment effect based on 10-year absolute risk reduction and lifetime benefit). QALYs were calculated by summing the time a person spent in a certain health state multiplied by the associated utility (supplemental table 2). A utility is a quality of life weight varying between 1.0 (perfect health) and 0.0 (death). In the present study, all patients start with a utility of 0.78, since all patients included have stable CVD. Utilities were derived from published data and measured with multi-attribute health status classification systems, mostly EQ-5D questionnaires [31–33]. Patients who experienced a revascularization were assumed to have the same quality of life as patients with stable CVD.

#### Costs

The costs of the cheapest available PCSK9-mAb (Alirocumab) in the Netherlands were taken as base case scenario [34]. Event costs and lifetime health care costs associated with vascular events were derived from observational studies in the Netherlands and from the Dutch nationwide registries. Lifetime costs made in the hospital, nursing home, and at the general practitioner were included [35–39]. Mean costs for a revascularization procedure were estimated as the weighted average for a PCI and a CABG [22]. Costs of pharmacist’s and laboratory tests for all patients were modelled. The cost of one extra doctor’s visit each year for prescription of PCSK9-mAbs was included. Costs in euros were updated to 2016 with the Dutch consumer price indices (supplemental table 2) [23].

#### Data analyses

The microsimulation model was run with a lifetime horizon for all 10,000 hypothetical patients within the described cohort for three different scenarios: (1) treat no one, (2) lifetime benefit-based treatment of the most eligible 5%, 10%, and 20% of patients, and (3) 10-year risk-based treatment of the most eligible 5%, 10%, and 20% of patients. Similar cut-off values were used to obtain equal numbers of treated patients. Mean costs, life years, and QALYs per patient were estimated for each of these scenarios and cut-off values. Incremental costs and QALYs were estimated for comparison between these scenarios. To calculate the incremental cost-effectiveness ratio (ICER), the incremental costs was divided by the incremental QALYs, expressed as costs spend per QALY gain.

#### Discount rates

Often, the costs and benefits considered in a health economic evaluation are not only incurred in the current year but materialize beyond the present. For the valuation of costs and benefits in the context of an economic evaluation, their timing is relevant because people generally value future costs and effects less than current costs and effects, and their value diminishes the more distant in the future they occur. Hence, economic evaluations need to adjust the value of costs and benefits for the time at which they occur, a technique known as discounting. For the Netherlands, standard discount rates of 4.0% for costs and 1.5% for health outcomes were applied [40, 41].

#### Exploration of the effects of uncertainties in the parameters

The dependence of the results on assumptions made for the model’s parameters were explored with analyses in which parameters such as drug costs, event probabilities, event costs, treatment effects of PCSK9-mAbs, discount rates, mortality multipliers, and utilities were varied for one-way and multi-way sensitivity analyses. The lower and upper bounds for the sensitivity analyses are shown in Supplemental table 1 for annual event risks and mortality and in Supplemental table 2 for costs and utilities.

The one-way scenario analyses were based on the treatment of 10% of the patients with the highest 10-year risk reduction and highest lifetime benefit.

For the multi-way probabilistic sensitivity analysis, a Monte Carlo simulation was performed 1000 times with all parameters being varied for each simulated person. For each simulation probability, hazard ratios for lowering LDL-c by PCSK9-mAbs, and utilities were randomly chosen from beta distributions. Mortality multipliers and costs were randomly chosen from gamma distributions. Individualized expected effects of PCSK9 inhibitors were calculated with the randomly chosen values for the parameters. The probability that risk-based and/or benefit-based treatment for different cut-off values would be cost-effective compared to no treatment with PCSK9-mAbs was displayed graphically for varying thresholds of the willingness to pay (in Euros) per QALY gained.

## Results

### Baseline characteristics

The baseline characteristics of our hypothetical study population of 10,000 patients are shown in Table 1. Patients selected for treatment based on the highest lifetime benefit are, on average, more than 10 years younger compared to patients selected based on the highest absolute 10-year CVD-risk reduction.

**Table 1:** Baseline characteristics

	All patients	% Patients with the highest expected lifetime benefit			% Patients with the highest expected 10-year risk reduction		
	*n* = 10,000	*n* = 500	*n* = 1000	*n* = 2000	*n* = 500	*n* = 1000	*n* = 2000
Age (years)	61 (8)	51 (5)	52 (5)	54 (6)	65 (8)	64 (8)	64 (8)
Male gender	7366 (74%)	274 (55%)	598 (60%)	1259 (63%)	346 (69%)	718 (72%)	1479 (74%)
Current smoking	3137 (31%)	116 (23%)	240 (24%)	531 (27%)	242 (48%)	471 (47%)	907 (45%)
Type 2 diabetes mellitus	1775 (18%)	46 (9%)	105 (11%)	210 (11%)	144 (29%)	280 (28%)	506 (25%)
Systolic blood pressure (mmHg)	140 (20)	142 (21)	141 (21)	140 (21)	150 (22)	148 (22)	146 (22)
Total cholesterol (mmol/L)	4.7 (4.0–5.6)	6.6 (5.9– 7.2)	6.2 (5.6–6.9)	5.9 (5.2–6.6)	6.9 (6.3–7.6)	6.4 (5.8–7.1)	6.0 (5.3–6.7)
Creatinine (umol/L)	89 (70–111)	90 (75–108)	90 (74–111)	90 (72–110)	107 (86–129)	106 (85–128)	103 (84–125)
1 location of CVD	7929 (79%)	414 (83%)	828 (83%)	1636 (82%)	284 (57%)	602 (60%)	1288 (64%)
2 location of CVD	1998 (20%)	86 (17%)	172 (17%)	359 (18%)	211 (42%)	386 (39%)	696 (35%)
3 location of CVD	73 (1%)	0 (0%)	0 (0%)	5 (0%)	5 (1%)	12 (1%)	16 (1%)
Coronary heart disease	6191 (62%)	314 (63%)	627 (63%)	1253 (63%)	339 (68%)	659 (66%)	1295 (65%)
Cerebrovascular disease	3110 (31%)	140 (28%)	299 (30%)	600 (30%)	155 (31%)	314 (31%)	663 (33%)
Peripheral artery disease	1924 (19%)	84 (17%)	163 (16%)	344 (17%)	130 (26%)	258 (26%)	467 (23%)
Abdominal aortic aneurysm	919 (9%)	48 (10%)	83 (8%)	172 (9%)	97 (19%)	179 (18%)	303 (15%)
Atrial fibrillation	278 (3%)	1 (0%)	3 (0%)	6 (0%)	32 (6%)	65 (7%)	106 (5%)
Chronic heart faillure	486 (5%)	7 (1%)	15 (2%)	42 (2%)	61 (12%)	117 (12%)	190 (10%)

All data are displayed as mean ± SD, median (Inter quartile range) or *n* (%)

*Locations of CVD: The number of locations of vascular disease (i.e. coronary heart disease, cerebrovascular disease, peripheral artery disease, or abdominal aortic aneurysm and combinations)

Treatment of the 5%, 10%, and 20% most eligible patients according to the lifetime benefit-based treatment strategy resulted in selection of patients with > 4.8 years, > 4.2 years, and > 3.5 years expected CVD life-years gain respectively. Treatment of the 5%, 10%, and 20% most eligible patients according to the 10-year risk-based treatment strategy resulted in selection of patients with > 12.3%, > 10.9% and > 9.2% expected 10-year absolute risk reduction of CVD, respectively. Seventy-two patients (14%) selected according to the 5% highest lifetime benefit-based treatment strategy were also selected according to the 5% highest 10-year risk-based treatment strategy. Two hundred patients (20%) selected according to the 10% highest lifetime benefit-based treatment strategy were also selected according to the 10% highest 10-year risk-based treatment strategy. Six hundred twelve patients (31%) selected according to the 20% highest lifetime benefit-based treatment strategy were also selected according to the 20% highest 10-year risk-based treatment strategy.

### Benefits

For each proportion threshold for treating (5%, 10%, and 20%), the groups treated on the basis of the lifetime benefit have, on average, higher QALYs than those on the basis of 10-year risk (Table 2).

**Table 2:** ICER for patients with the highest lifetime benefit-based treatment estimates and the highest 10-year risk-based treatment estimates

Cut-off value	5% of patients treated			10% of patients treated			20% of patients treated		
	Costs	QALYs	ICER	Costs	QALYs	ICER	Costs	QALYs	ICER
**No treatment**	€111,727,411	65,897	-	€111,732,871	65,935	-	€111,620,058	65,893	-
**Lifetime benefit-based treatment**	€153,997,681	67,057	€36,440/QALY	€194,164,605	68,014	€39,650/QALY	€269,495,928	69,704	€41,426/QALY
**10-year risk-based treatment**	€153,071,526	66,755	€48,187/QALY	€192,212,131	67,443	€53,368/QALY	€266,326,909	68,846	€52,390/QALY

Costs and QALYs are given for the scenario of 10,000 patients

*QALYs* quality-adjusted life years, *ICER* incremental cost-effectiveness ratio

**Fig. 3:**
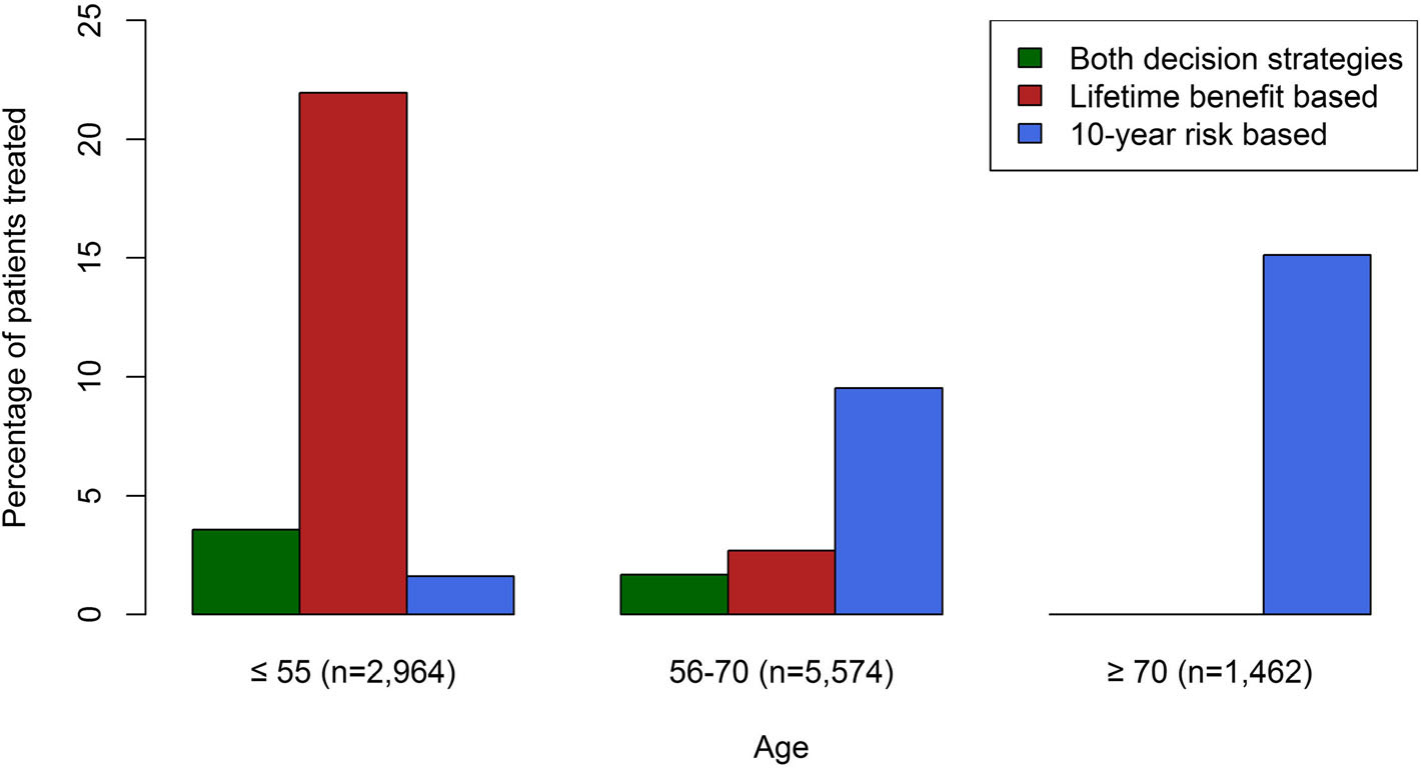
A histogram of the numbers of patients identified for treatment using the lifetime benefit-based strategy and the 10-year risk-based strategy stratified for age groups (< 55, 56–70, > 70)

Also, a higher number of younger patients were identified as treatment candidates on the basis of lifetime benefit than on the basis of 10-year risk (Fig. 3).

### Cost-effectiveness

Treatment decisions (treatment with a PCSK9-mAb versus no treatment) for each proportion threshold for treating (5%, 10%, and 20%) the groups treated on the basis of lifetime benefit have, on average, lower ICERs than those treated on the basis of 10-year risk (Table 2).

### Uncertainty analyses

The one-way sensitivity analysis found that therapy becomes less cost-effective if CVD event rates are lower than assumed and more cost-effective if CVD event rates are higher. If therapy is less expensive, on average, treatment becomes more cost-effective, while with more expensive therapy, on average, treatment becomes less cost-effective.

**Fig. 4:**
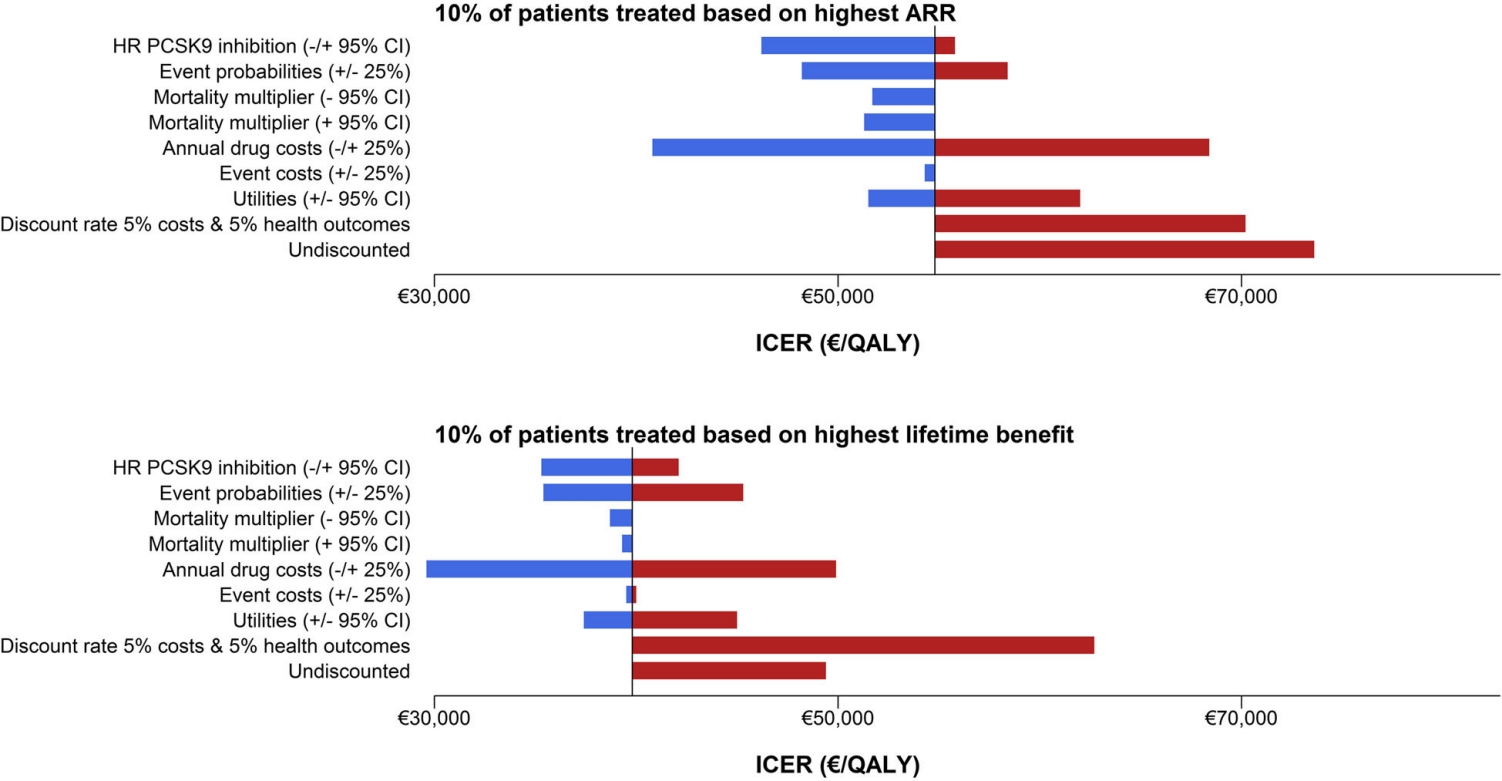
Scenario analyses estimating the influence of different model assumptions on **a**) the ICER of the lifetime benefit-based treatment strategy of 10% of the patients vs. no treatment and **b**) the ICER of the 10-year risk-based treatment strategy of 10% of the patients vs no treatment

**Fig. 5:**
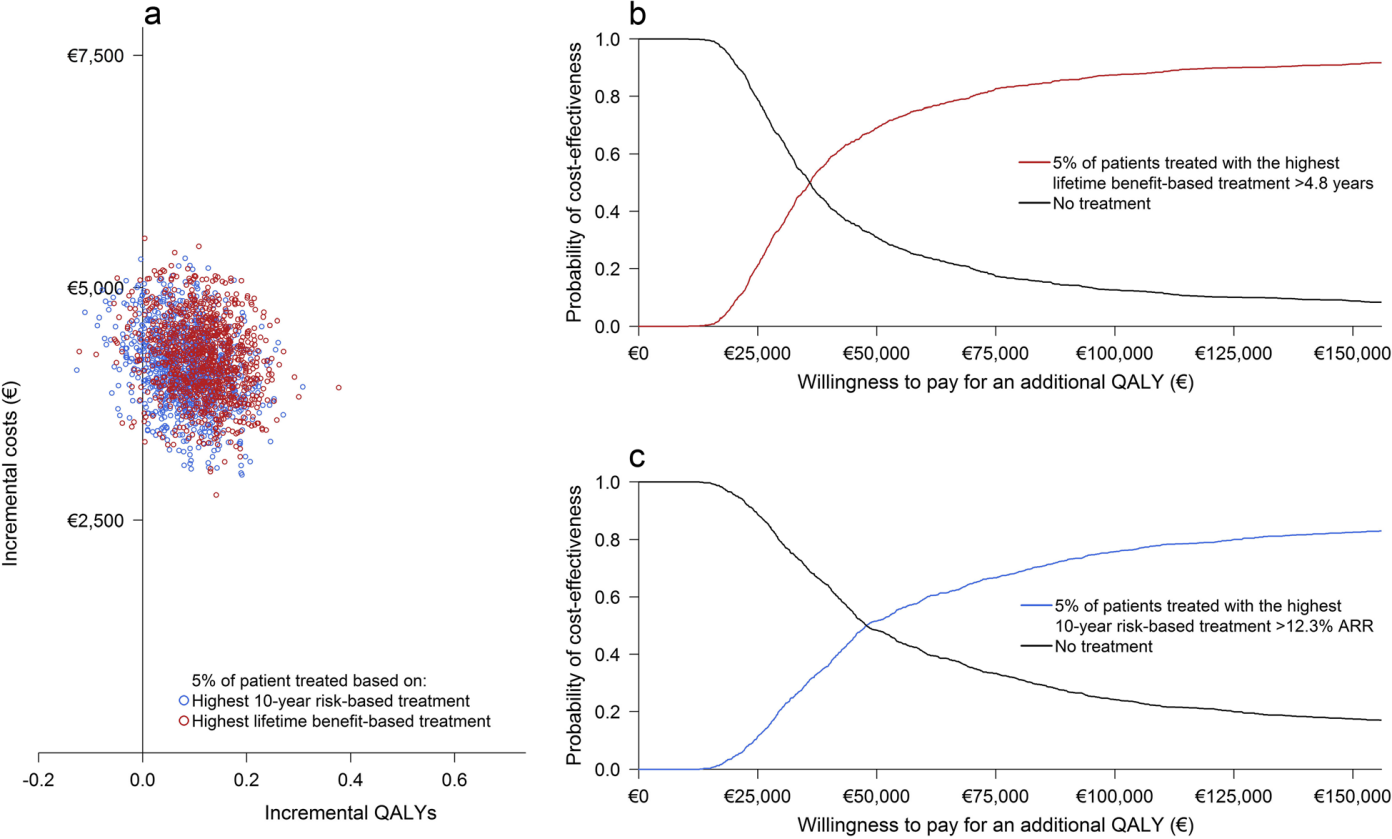
Incremental cost-effectiveness plane of the lifetime benefit-based strategy and the 10-year risk-based treatment strategy for 5% of patients treated with PCSK9-mAbs (**a**). Additional cost-effectiveness acceptability curves for both strategies separately (**b** lifetime benefit-based treatment, **c** 10-year risk-based treatment)

**Fig. 6:**
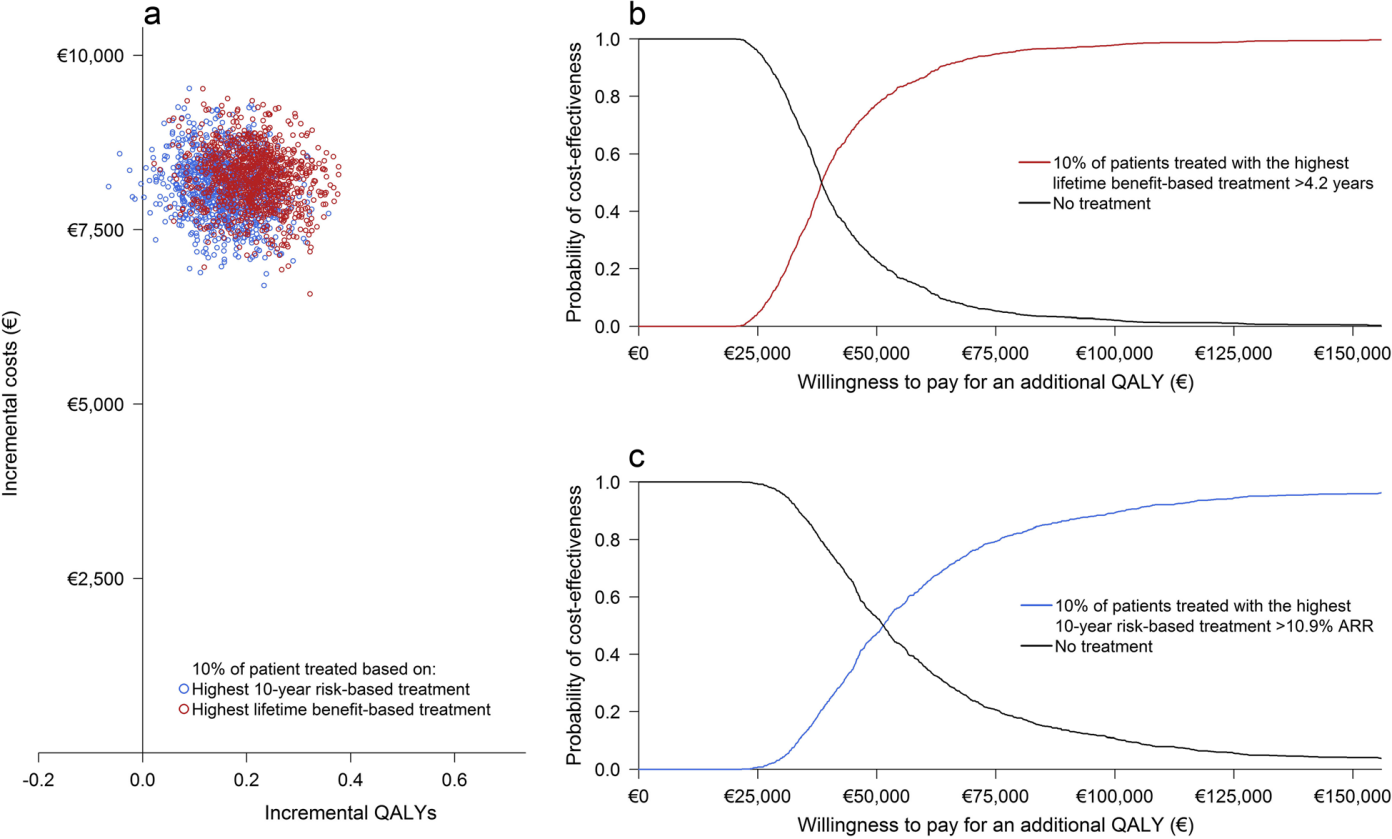
Incremental cost-effectiveness plane of the lifetime benefit-based strategy and the 10-year risk-based treatment strategy for 10% of patients treated with PCSK9-mAbs (**a**). Additional cost-effectiveness acceptability curves for both strategies separately (**b** lifetime benefit-based treatment, **c** 10-year risk-based treatment)

**Fig. 7:**
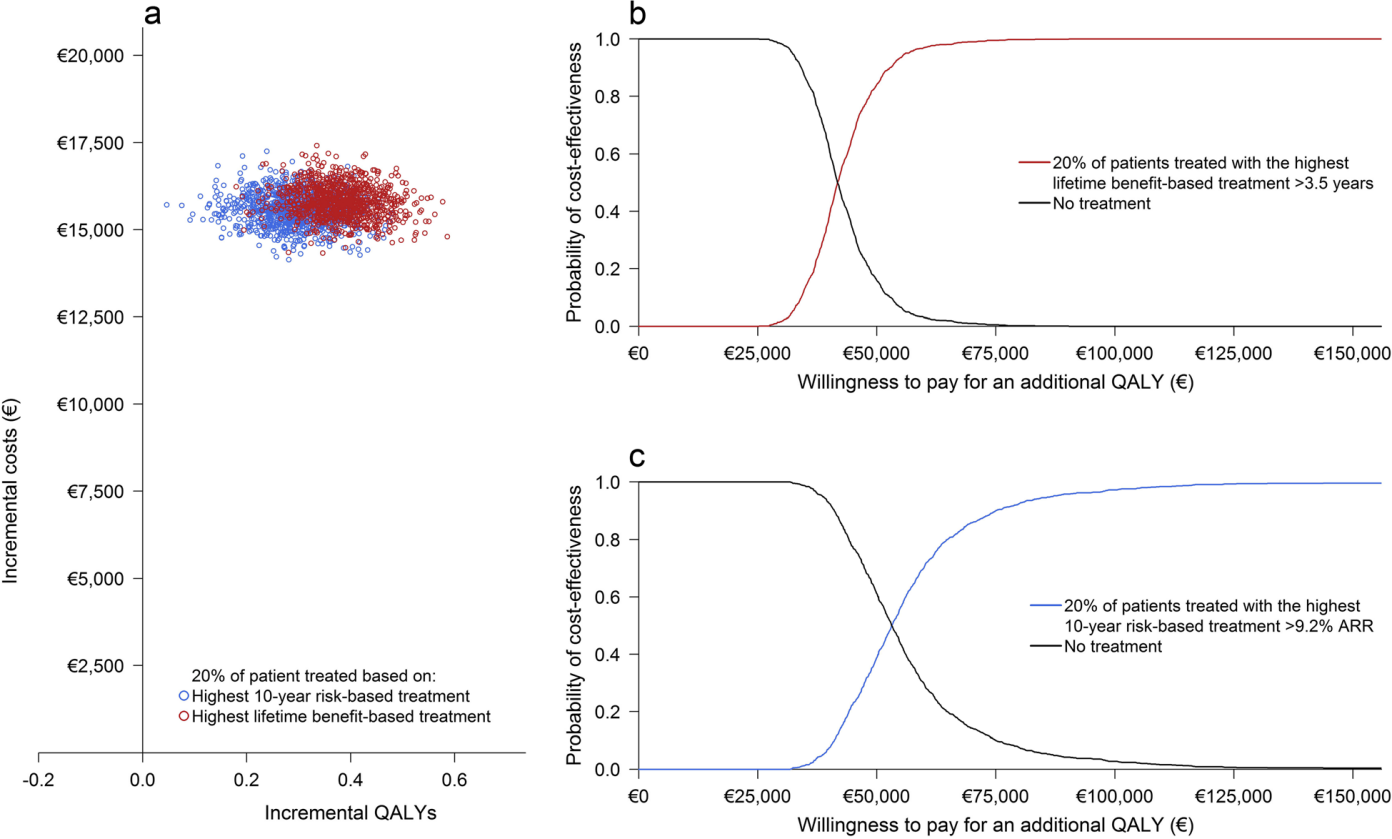
Incremental cost-effectiveness plane of the lifetime benefit-based strategy and the 10-year risk-based treatment strategy for 20% of patients treated with PCSK9-mAbs (**a**). Additional cost-effectiveness acceptability curves for both strategies separately (**b** lifetime benefit-based treatment, **c** 10-year risk-based treatment)

A 5% higher or lower discount for both costs and health outcomes and undiscounted analyses showed an increase in ICER for both strategies (Fig. 4).The multi-way probability sensitivity analyses found that treatment with PCSK9-therapy is, on average, always more expensive than no treatment at all. The probability of treatment being cost-effective therefore depends on the willingness to pay; generally, €50,000 per additional QALY is considered acceptable [42]. For this level of willingness to pay, the probability that lifetime benefit-based treatment of 5%, 10%, and 20% of patients is cost-effective compared to no treatment at all is 69.0%, 77.2%, and 84.1% respectively (Figs. 5, 6, and 7; Table 3). Similarly, the probability that the 10-year risk-based treatment of the 5%, 10%, and 20% most eligible patients is cost-effective compared to no treatment at all is 51.6%, 47.3%, and 38.8% respectively (Figs. 5, 6, and 7; Table 3). The level of willingness to pay, however, can be debated. The lower bound of willingness to pay for which treatment is > 50% certain cost-effective was, on average, €35,900/QALY, €38,400/QALY, and €41,700/QALY for 5%, 10%, and 20% most eligible patients based on the lifetime benefit-based treatment strategy and on average €47,800/QALY, €51,300/QALY, and €53,100/QALY for 5%, 10%, and 20% most eligible patients based on the 10-year risk-based treatment strategy (Table 3).

**Table 3:** Percentage of multi-way probability sensitivity analyses that are cost-effective for a willingness to pay €50,000 per QALY and the lower bound of willingness per QALY in euros for which 50% of the multi-way probability analyses are cost-effective

Cut-off value	5% of patients treated	10% of patients treated	20% of patients treated
Willingness to pay €50,000 per additional QALY			
Lifetime benefit based treatment	69%	77.2%	84.1%
10-year risk-based treatment	51.6%	47.3%	38.8%
Lower bound of willingness to pay per additional QALY			
Lifetime benefit based treatment	€ 35,900	€ 38,400	€ 41,700
10-year risk-based treatment	€ 47,800	€ 51,300	€ 53,100

## Discussion

In the present study, it is shown that treatment decisions are both more effective and more cost-effective when based on estimated lifetime benefit than when based on estimated risk reduction over 10 years, at least in this illustration example of PSCK9 inhibition in patients with stable CVD. Although the results are sensitive to the assumptions made, our uncertainty analyses show that the lifetime benefit-based treatment strategy remained favourable compared to the traditional absolute risk reduction-based treatment strategy in all one-way and multi-way probability sensitivity analyses.

Increasing evidence suggests that estimation of lifetime benefit may help to identify a group of patients with previously underappreciated long-term potential for benefiting from preventive treatment. A large pooled survival analysis with more than 900,000 person-years using data from 5 community-based cohort studies from 1964 through 2008 showed that individuals with an index age of 45 with at least 2 risk factors lose, on average, 14 life-years free of CVD compared to individuals with optimal risk factor profiles. The loss in life-years free of CVD for individuals with an index age of 75 was only 4 years, compared to individuals with optimal risk factors. This suggests that long-term exposure to risk factors at younger age has more impact on life-years lost, despite the fact that 10-year CVD-risk is still low [43, 44].

These findings are also in line with a modelling study on aspirin use in healthy women [5]. That study showed that aspirin use is associated with the highest lifetime benefit in younger women with otherwise high risk factor levels. In contrast, the women with the highest 10-year CVD-risk, who were generally older, experienced a lower lifetime benefit of aspirin use to prevent CVD. It was suggested that treatment decision-making for the highest treatment effect based on lifetime benefit improved health outcomes [45–47]. In a microsimulation based on a population-based cohort of individuals aged 55 years and older, it has been shown that the youngest individuals with high risk factor levels have the highest CVD-free gain in life expectancy with statin therapy [47]. However, based on these studies, the question remains whether improvements in health outcomes would outweigh the costs of longer treatment duration in these younger patients.

Our findings provide evidence that the improved health outcomes due to treatment decisions based on the highest lifetime benefit do outweigh the costs of longer treatment duration compared to treatment decisions based on the highest risk reduction. The use of lifetime benefit estimates identified younger patients that would not be treated based on their 10-year risk predictions. This raises the question whether or not lifetime benefit-based strategies should be recommended for other treatments and in other patient populations. The uncertainty analyses estimating the influence of different assumptions in the model give a sense of changing parameters, but it is merely speculating whether the lifetime benefit-based treatment strategy is superior to the 10-year risk-based treatment strategy in other settings. Whether the cost-effectiveness of the lifetime benefit-based treatment decisions are generalizable in other populations, for instance in a primary prevention setting, should be established in other studies.

Also the use of other 10-year risk models could change the results of this study. However, it seems unlikely that this would lead to a different conclusion. Unlike most other 10-year risk models, the SMART-REACH model takes competing risks into account, preventing overestimation of risk and treatment effect in older patients. Thus, using different 10-year risk models without adjustment for competing risk would probably lead to higher misclassification of older individuals as treatment candidates and, therefore, result in even higher benefit of using a lifetime prediction model.

Additionally, before lifetime benefit can be used to guide clinical decision-making in other settings, thresholds at which treatment is recommended should be investigated. For a specific preventive intervention, a cost-effectiveness analysis can be performed to establish a threshold of disease-free lifetime benefit gained at which an intervention is cost-effective. For PCSK9 inhibition in a population with stable CVD and a willingness to pay €50,000 per additional QALY, lifetime benefit-based treatment is cost-effective for patients with a lifetime benefit of > 3.1 years.

Strengths of this study include the use of the microsimulation model, in which a cohort of patients can be exposed to multiple strategies with a lifetime horizon. It also made it possible to simulate the effect of multiple strategies for individual patients instead of simulations on a population level. Also, we based our assumptions on recent peer-reviewed literature and adjusted event probabilities and risk of death for the age and cardiovascular history of patients. Furthermore, we performed various scenario analyses and sensitivity analyses that showed the effect of assumption on the cost-effectiveness.

Some limitations should be considered. First, in this cost-effectiveness analysis, we used PCSK9 inhibitors as an example. It is unsure whether our results are generalizable for other treatments. Hypothetically, selection of patients based on lifetime benefit is even more cost-effective compared to 10-year risk-based selection for less expensive treatments with similar efficacy, for example statins. However, longer treatment duration with different medication also results in more adverse effects. It is merely a speculation whether this outweigh the potential gain in QALYs. It would be reassuring to find similar results with for example statin therapy in primary prevention setting, statin therapy in patients with diabetes mellitus, or PCSK9 inhibition in patients with familiar hypercholesterolemia. Secondly, it should be noted that the results are based on a simplified model. For example, we only modelled the effect of treatment on first recurrent events but not on subsequent ones. This might have led to slight underestimation of the benefit of treatment of both strategies. It would be reassuring if other more complex modelling studies were to show similar results in favour of the lifetime benefit strategy. Also, in this study, the assumption was made that there is a 100% adherence to therapy. In clinical practice, this is not true. However, between the two strategies to identify patients that would benefit from treatment, the adherence rate will probably be similar, and therefore, it would not change the difference of effects of treatment between the two decision strategies. Third, the possibility to postpone treatment to an older age was not taken into account. For the selection of patients with the highest lifetime benefit, the possibility to postpone treatment will not influence the selection, since the life-year gained only decreases with postponed treatment. For the selection of patients with the highest 10-year risk reduction, there could be a difference in patient selection due to increasing 10-year risk with age, and aligned with that an increased 10-year risk reduction. However, the results of our study would not be different, since the utility gain and the costs for treatment in patients with a postponed treatment will be similar to the relative older patients selected at the start of the simulation.

Finally, the harm and disutility of PCSK9 mAbs was not incorporated in the model. Assuming that harm is independent of the cardiovascular risk and benefit of the treatment, this would be similar for the patients treated based on the highest lifetime benefit and the patients treated based on the highest absolute risk reduction. However, patients with the highest lifetime benefit are treated for a longer duration. Since PCSK9-mAbs are a new class of drugs, there is limited information on the harm of PCSK9 inhibition, especially in the long run. Therefore the microsimulation analyses should be re-adjusted including harm of treatment whenever any risk of harm is observed.

For future work, we would recommend developing more realistic models for primary and secondary prevention of CVD and for other clinical applications that take into account competing risks and enable lifetime estimations of individual risk and benefit of preventive treatment.

## Conclusions

Treatment decisions (treatment with a PCSK9-mAb versus no treatment) are both more effective and more cost-effective when based on estimated lifetime benefit than when based on estimated risk reduction over 10 years.

## Supplementary information

**Additional file 1: **Supplementary appendix REACH-SMART model^1^. **Supplemental table 1.** Annual event risks and mortality multipliers. **Supplemental table 2.** Costs and utilities. **Supplemental figure 1.** Age-adjusted annual event rates in percentage.

## Data Availability

Raw clinical data and codes used to create a hypothetical study population for the simulation and the simulations itself will be available on request at the corresponding author.

## Abbreviations

CABG: Coronary arterial bypass grafting
CVD: Cardiovascular disease
HRs: Hazard ratios
ICER: Incremental cost-effectiveness ratio
LDL-c: Low-density lipoprotein cholesterol
mAbs: Monoclonal antibodies
MACE: Major cardiovascular events
PCI: Percutaneous coronary intervention
PCSK9: Proprotein convertase subtilisin/kexin type 9
QALYs: Quality-adjusted life years
SMART-REACH model: A prediction model able to estimate individual benefit of medication for prevention of CVD in patients with a history of stable CVD as 10-year risk reduction or as months gained from a lifetime perspective
SMART cohort: Second Manifestation of ARTerial disease cohort, a cohort of patients with a history of stable CVD
