# Supplementary material for: Would treatment decisions about secondary prevention of CVD based on estimated lifetime benefit rather than 10-year risk reduction be cost-effective?
Source: Diagn Progn Res. 2020 Apr 16;4:4. doi: 10.1186/s41512-020-00072-5 (PMC7161238; doi:10.1186/s41512-020-00072-5)
Supplement: Supplementary file 1 — Additional file 1: Supplementary appendix REACH-SMART model1. Supplemental table 1. Annual event risks and mortality multipliers. Supplemental table 2. Costs and utilities. Supplemental figure 1. Age-adjusted annual event rates in percentage. [file 41512_2020_72_MOESM1_ESM.docx]

**Supplementary appendix**

*REACH-SMART model^1^*

The REACH-SMART model is a prediction model with a lifetime horizon. It has been developed in the REduction of Atherothrombosis for Continued Health (REACH) and external validated in the Secondary Manifestations of ARTerial disease (SMART) cohort. REACH and SMART are prospective cohort studies of patients with clinical vascular disease or vascular risk factors. Study details have been described elsewhere.^2 3^ The statistical methods of the model were previously described in detail.^4 5^ In short, two Fine and Gray competing risk models were fitted for cause specific estimates of the cumulative incidence, one for recurrent vascular events (stroke, MI, or vascular death) and one for non-vascular mortality. Age was used as the underlying time function (i.e., left-truncation). This enables lifetime predictions across the age range from the youngest age at study entry to the highest age at study exit. Predictors were pre-specified based on existing prediction models and on availability in both datasets. Nine predictors were used for both Fine and Gray models: sex, current smoking (yes/no), diabetes mellitus (yes/no), systolic blood pressure (mmHg), total cholesterol (mmol/L), creatinine (umol/L), number of locations of vascular disease (i.e., CAD, CVD, and PAD), history of atrial fibrillation (yes/no) and history of congestive heart failure (yes/no).

Beginning at the starting age of each individual, the cumulative survival free of myocardial infarction (MI) and stroke was estimated for each subsequent year. The estimated survival free of MI and stroke at the beginning of each life-year was multiplied by the survival probability during that year. The survival probability was obtained by subtracting vascular risk and non-vascular mortality risk from one.

Life-expectancy free of stroke or MI of an individual person was defined as the median estimated survival, which is the age where the predicted individual survival curve equals 50%. The REACH-SMART model can estimate 10-year CVD-risks, by truncating cause-specific estimates of vascular risk at 10 years after the starting age. An individual’s benefit from lifelong treatment was estimated as the difference between the estimated survival with and without treatment.

|  | Base  case | Lower bound | Upper bound | Source | Reference |
| --- | --- | --- | --- | --- | --- |
| Mean annual event risk*(%) |  |  |  |  |  |
| Myocardial infarction | 1.26 |  |  | Observational study | ^3^ |
| Stroke | 0.66 |  |  | Observational study | ^3^ |
| Resuscitated cardiac arrest | 0.10 |  |  | RCT | ^6^ |
| Revascularization | 2.23 |  |  | Observational study | ^3^ |
| Chronic heart failure | 0.49 |  |  | RCT | ^6^ |
|  |  |  |  |  |  |
| Mortality multipliers |  |  |  |  |  |
| Stable CVD |  |  |  |  |  |
| Coronary artery disease | 2.5 | 2.3 | 2.7 | Observational study | ^7^ |
| Cerebrovascular disease | 2.3 | 2.0 | 2.7 | Observational study | ^8^ |
| Peripheral artery disease | 3.1 | 1.9 | 4.9 | Observational study | ^9^ |
| Abdominal aortic aneurysm | 1.7 | 1.6 | 1.8 | Observational study | ^10^ |
| Myocardial infarction | 2.4 | 2.1 | 2.7 | Observational study | ^3^ |
| Stroke | 1.9 | 1.6 | 2.2 | Observational study | ^3^ |
| Resuscitated cardiac arrest | 2.4 | 2.1 | 2.7 | Observational study | ^3^ |
| Revascularization | 1.6 | 1.3 | 2.1 | Observational study | ^11^ |
| Chronic heart failure | 2.1 | 1.8 | 2.7 | Observational study | ^12^ |
|  |  |  |  |  |  |

**Supplemental table 1.** Annual event risks and mortality multipliers.

*Mean annual event risk is the risk for a 60 year old patient.

**Supplemental table 2.** Costs and utilities.

|  | Base  Case | Lower  bound | Upper bound | Source | Reference |
| --- | --- | --- | --- | --- | --- |
| Costs |  |  |  |  |  |
| Drug (annual costs for 1 patient) |  |  |  |  |  |
| PCSK9-mAbs | € 5,981 | € 4,486 | € 7,476 | Official tariff | ^13^ |
| Event |  |  |  |  |  |
| Myocardial infarction | € 5,037 | € 3,778 | € 6,296 | Observational study | ^14^ |
| Stroke | € 19,030 | € 14,273 | € 23,788 | Dutch registries | ^15^ |
| Resuscitated cardiac arrest | € 28,636 | € 21,477 | € 35,795 | Observational study | ^16^ |
| Revascularization | € 6,944 | € 5,009 | € 8,349 | Observational study | ^14 16 17^ |
| Post-event care |  |  |  |  |  |
| Stroke | € 9,827 | € 7,370 | € 12,284 | Dutch registries | ^15^ |
| Chronic heart failure | € 6,569 | € 4,927 | € 8,211 | Dutch registries | ^15^ |
| Other costs |  |  |  |  |  |
| Doctor's visit | € 109 | € 69 | € 157 | Official tariff | ^18^ |
| Pharmacy | € 26 | € 11 | € 52 | Official tariff | ^18^ |
| Laboratory | € 25 | € 17 | € 37 | Official tariff | ^18^ |
| Utilities |  |  |  |  |  |
| Stable CVD | 0.78 | 0.69 | 0.83 | Observational study | ^19^ |
| Myocardial infarction | 0.65 | 0.56 | 0.70 | Observational study | ^19^ |
| Stroke | 0.64 | 0.55 | 0.69 | Observational study | ^19^ |
| Resuscitated cardiac arrest | 0.65 | 0.42 | 0.75 | Observational study | ^20^ |
| Chronic heart failure | 0.63 | 0.51 | 0.72 | Observational study | ^21^ |

**Supplemental figure 1.** Age-adjusted annual event rates in percentage.


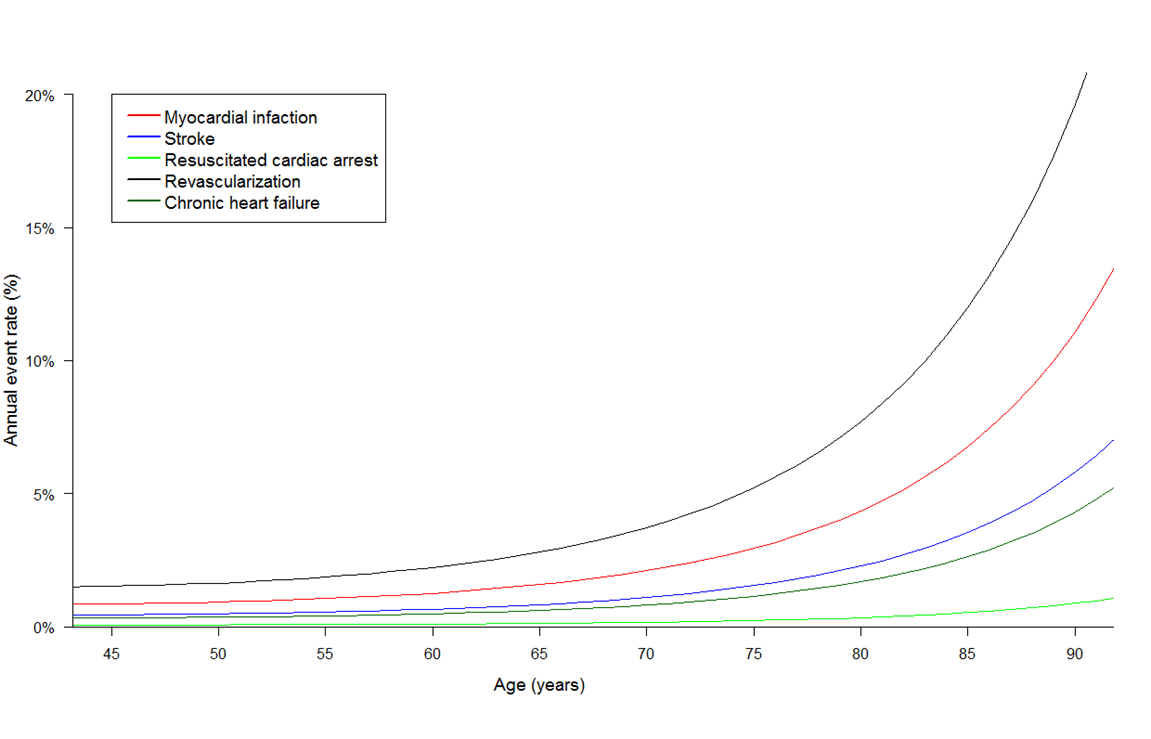


**References**

1. Kaasenbrood L, Bhatt DL, Dorresteijn JAN, et al. Estimated Life Expectancy Without Recurrent Cardiovascular Events in Patients With Vascular Disease: The SMART‐REACH Model. Journal of the American Heart Association 2018.

2. Bhatt DL, Steg PG, Ohman EM, et al. International prevalence, recognition, and treatment of cardiovascular risk factors in outpatients with atherothrombosis. Jama 2006;**295**(2):180-9.

3. Simons PC, Algra A, van de Laak MF, et al. Second manifestations of ARTerial disease (SMART) study: rationale and design. European journal of epidemiology 1999;**15**(9):773-81.

4. Dorresteijn JAN, Kaasenbrood L, Cook NR, et al. How to translate clinical trial results into gain in healthy life expectancy for individual patients. Bmj-Brit Med J 2016;**352**.

5. Geskus RB. Cause-specific cumulative incidence estimation and the fine and gray model under both left truncation and right censoring. Biometrics 2011;**67**(1):39-49.

6. LaRosa JC, Grundy SM, Waters DD, et al. Intensive lipid lowering with atorvastatin in patients with stable coronary disease. The New England journal of medicine 2005;**352**(14):1425-35.

7. Bronnum-Hansen H, Jorgensen T, Davidsen M, et al. Survival and cause of death after myocardial infarction: the Danish MONICA study. J Clin Epidemiol 2001;**54**(12):1244-50.

8. Dennis MS, Burn JP, Sandercock PA, et al. Long-term survival after first-ever stroke: the Oxfordshire Community Stroke Project. Stroke; a journal of cerebral circulation 1993;**24**(6):796-800.

9. Criqui MH, Denenberg JO, Langer RD, et al. The epidemiology of peripheral arterial disease: importance of identifying the population at risk. Vasc Med 1997;**2**(3):221-6.

10. Eldrup N, Budtz-Lilly J, Laustsen J, et al. Long-term incidence of myocardial infarct, stroke, and mortality in patients operated on for abdominal aortic aneurysms. Journal of vascular surgery 2012;**55**(2):311-7.

11. Komocsi A, Simon M, Merkely B, et al. Underuse of coronary intervention and its impact on mortality in the elderly with myocardial infarction. A propensity-matched analysis from the Hungarian Myocardial Infarction Registry. International journal of cardiology 2016;**214**:485-90.

12. Mosterd A, Cost B, Hoes AW, et al. The prognosis of heart failure in the general population: The Rotterdam Study. European heart journal 2001;**22**(15):1318-27.

13. Dutch Health Care Insurance Board. Drug costs 2016. [www.medicijnkosten.nl](http://www.medicijnkosten.nl). Accessed on: 22-09-2016.

14. Soekhlal RR, Burgers LT, Redekop WK, et al. Treatment costs of acute myocardial infarction in the Netherlands. Neth Heart J 2013;**21**(5):230-5.

15. Dutch nationwide registries. [www.kostenvanziekten.nl](http://www.kostenvanziekten.nl). Accessed on: 22-09-2016.

16. Ringborg A, Nieuwlaat R, Lindgren P, et al. Costs of atrial fibrillation in five European countries: results from the Euro Heart Survey on atrial fibrillation. Europace 2008;**10**(4):403-11.

17. van Mastrigt GA, Heijmans J, Severens JL, et al. Short-stay intensive care after coronary artery bypass surgery: randomized clinical trial on safety and cost-effectiveness. Crit Care Med 2006;**34**(1):65-75.

18. Greving JP, Visseren FL, de Wit GA, et al. Statin treatment for primary prevention of vascular disease: whom to treat? Cost-effectiveness analysis. BMJ 2011;**342**:d1672.

19. Sullivan PW, Lawrence WF, Ghushchyan V. A national catalog of preference-based scores for chronic conditions in the United States. Med Care 2005;**43**(7):736-49.

20. Deasy C, Bray J, Smith K, et al. Functional outcomes and quality of life of young adults who survive out-of-hospital cardiac arrest. Emerg Med J 2013;**30**(7):532-7.

21. Salomon JA, Vos T, Hogan DR, et al. Common values in assessing health outcomes from disease and injury: disability weights measurement study for the Global Burden of Disease Study 2010. Lancet 2012;**380**(9859):2129-43.
